# Association between Secondary and Primary Sjögren's Syndrome in a Large Collection of Lupus Families

**DOI:** 10.1155/2015/298506

**Published:** 2015-07-12

**Authors:** Rachna Aggarwal, Juan-Manuel Anaya, Kristi A. Koelsch, Biji T. Kurien, R. Hal Scofield

**Affiliations:** ^1^Department of Medicine, University of Oklahoma Health Sciences Center, Oklahoma City, OK 73104, USA; ^2^Arthritis & Immunology Program, Oklahoma Medical Research Foundation, Oklahoma City, OK 73104, USA; ^3^Center for Autoimmune Diseases Research (CREA), Universidad del Rosario, Bogotá, Colombia; ^4^Medical Service, Department of Veterans Affairs Medical Center, Oklahoma City, OK 73104, USA

## Abstract

*Objective*. Systemic lupus erythematosus (SLE) and Sjögren's syndrome (SS) share clinical and immunogenetic features and may occur together. We undertook this study to determine the risk of primary SS among SLE-unaffected relatives of SLE patients and whether or not primary and secondary SS tended to occur in the same families. *Methods*. We collected clinical and serological data on 2694 SLE patients, 7390 SLE-unaffected relatives of the SLE patients, and 1470 matched controls. *Results*. Of the 2694 subjects with SLE, 548 had secondary SS, while 71 of their 7390 SLE-unaffected relatives had primary SS. None of the 1470 controls had SS as defined herein (*p* = 5 × 10^−5^ compared to SLE-unaffected relatives). Of the 71 SLE-unaffected relatives with primary SS, 18 (25.3%) had an SLE-affected family member with secondary SS, while only 530 of the 7319 (7.2%) SLE-unaffected relatives without SS did so (*p* = 1 × 10^−8^). *Conclusion*. Among families identified for the presence of SLE, primary and secondary SS tend to occur within the same families. These results highlight the commonalities between these two forms of SS, which in fact correspond to the same disease.

## 1. Introduction

Sjögren's syndrome (SS) is a chronic autoimmune disease that characteristically affects salivary and lacrimal glands such that patients have severe dry eyes and dry mouth [1]. Pathology in these glands consists of lymphocytic infiltration, commonly with germinal center formation [2]. The disease may also involve numerous other organs, including the lungs, kidneys, joints, skin, peripheral nerves, and brain. Patients with SS are at high risk for mucosal-associated lymphoid tissue (MALT) lymphoma [3]. The diagnosis of SS is based on the combination of symptoms (sicca symptoms) and the presence of the autoimmune characteristics: activation of T cells (confirmed in practice by a positive salivary gland biopsy) or B cells (i.e., presence of autoantibodies). The most common specificities are anti-Ro (or SSA) and anti-La (or SSB) [4]. SS may be the second only to rheumatoid arthritis in terms of prevalence of the inflammatory rheumatic illnesses [5].

In most of the cases, SS occurs without any other inflammatory, autoimmune illness. In this case, the disease is still termed primary. Meanwhile, SS can also occur in association with another autoimmune disease (i.e., polyautoimmunity), the most frequent being autoimmune thyroid disease, rheumatoid arthritis (RA), and systemic lupus erythematosus (SLE) [6]. When this occurs, the disease is designated secondary. The classification criteria for SS are those of the American-European Consensus Group (AECG), which require one of either salivary gland pathology showing foci of lymphocytic infiltration or positive serology in the form of anti-Ro or anti-La [7]. Recently, new criteria have been proposed [8] and compared to the AECG criteria [9].

Among those with SLE, about 20% of patients have secondary SS. SLE and SS can share clinical features such as nondeforming, inflammatory arthritis, photosensitivity, and lymphopenia [10]. In addition, anti-Ro and anti-La are also found in the sera of patients with SLE, although at a lower rate than in SS [7].

Risk of SLE and SS clearly has a genetic component [11]. However, the study of the genetics of SLE is much farther along than that of SS. There are now multiple genome-wide association studies reported for the former with genetic association reported and confirmed for many dozens of genes [12]. On the other hand, two genome-wide association studies have been reported for SS and show some associations in common with SLE while others are unique to SS [13, 14]. Other reported genetic associations for SS are from small cohorts in which candidate genes already known to be associated with SLE were studied [11].

We have collected a large cohort of patients with SLE and have data concerning the presence of sicca symptoms, autoantibodies, and a past diagnosis of SS in both the SLE patients and their SLE-unaffected relatives. Thus, we undertook this study to determine whether there is a familial relationship between putative “primary” and “secondary” SS.

## 2. Methods

### 2.1. Lupus Family Registry and Repository

We collected data and biological samples from families with SLE for over two decades. The methods of this resource have been presented in depth [15]. Briefly, putative SLE patients completed an extensive questionnaire, underwent an interview, and had medical records reviewed to ensure that all patients met the American College of Rheumatology SLE classification criteria [10, 16]. Family members also completed a questionnaire about their health status and were interviewed by trained personnel if there were any answers suggesting a diagnosis of SLE. Unrelated controls were also recruited and by questionnaire and interview were determined not to have SLE, as defined by classification criteria.

### 2.2. Serology

All subjects underwent serological testing for SLE-associated autoantibodies in the Oklahoma Medical Research Foundation Clinical Immunology Laboratory, a CLIA-approved facility. For purposes of the present study, this included determination of anti-Ro60 and anti-La by double immunodiffusion [17, 18].

### 2.3. Putative Sjögren's Syndrome

SS was defined in part by the presence of dry mouth and dry eye symptoms. All subjects, both SLE patients and their SLE-unaffected relatives, were asked the following questions: (1) have you had daily, persistent, troublesome dry eyes for more than three months?; (2) do you have a recurrent sensation of sand or gravel in the eyes?; (3) do you use tear substitutes more than three times a day?; (4) have you had a daily feeling of dry mouth for more than three months?; (5) have you had recurrently or persistently swollen salivary glands as an adult?; (6) do you frequently drink liquids to aid in swallowing dry foods?; and (7) have you ever been told by a doctor that you have Sjögren's syndrome? The first six questions allow classification as to presence or absence of the American-European Consensus Group SS classification criteria for subjective dry eyes and dry mouth [7]. These questions also correspond to those asked in the Connective Disease Screening Questionnaire, a validated tool with high sensitivity and specificity for SS [19]. Thus, SLE patients were classified as “secondary” SS if dry eyes, dry mouth, and anti-Ro were present. SLE-unaffected relatives were classified as “primary” SS if they gave a history of a diagnosis of the disease or if at least one dry eye and at least one dry mouth were answered positively and anti-Ro was present.

### 2.4. Statistics

Categorical data were compared by a chi square analysis. A *p* value less than 0.05 was considered statistically significant.

## 3. Results

A total of 2694 subjects meeting the ACR SLE classification criteria [10, 16] were considered in the analyses. There were 2400 (89.1%) women and 294 (10.9%) men. Among these, 45.2% self-identified as white Americans, 36.4% as black Americans, 9.3% as Hispanic Americans, 0.4% as Asian Americans, and 6% as Native Americans. There were 7390 relatives of these lupus patients who entered the study, none of whom met these same criteria for the classification of SLE. There were 2459 (33.3%) male relatives and 4931 (66.7%) female relatives. The unrelated controls numbered 1470 and were matched for age, sex, and ethnicity/race to the SLE patients.

Among the 2694 SLE patients, there were 548 (20.3%) who had dry eyes, dry mouth, and anti-Ro, that is, putative “secondary” SS as we have defined it. Of these, 507 (92.5%) were women. A plurality, 228 (41%), was white Americans, with 218 (39.7%) black, 3 (0.5%) Asian, 45 (8.2%) Hispanic, and 35 (6.3%) Native Americans. Among the 7390 SLE-unaffected relatives, there were 71 who met our criteria for putative SS. None of the 1470 unrelated controls met these criteria, which was less common compared to the SLE-unaffected relatives (*χ* ^2^ = 16.8, *p* = 0.00004).

We then considered the distribution of “primary” and “secondary” SS among the subjects with SLE as well as their SLE-unaffected relatives. Of the 548 with “secondary” SS, 18 had a relative with “primary” SS. Meanwhile, of those with SLE but no “secondary” SS, only 53 of 2146 had a non-SLE-affected relative with “primary” SS (*χ* ^2^ = 1.3, *p* = NS, odds ratio = 1.4). Thus, SLE patients with “secondary” SS were about ~1.5 times more likely to have a family member with “primary” SS, compared to those SLE-affected subjects without “secondary” SS, but this difference did not reach statistical significance.

Alternatively, we considered the presence of “secondary” SS within the families with “primary” SS. There were 71 “primary” SS patients, of whom 18 (25.3%) had a relative with “secondary” SS, while, among 7319 lupus-unaffected relatives without SS, 530 (7.1%) had a lupus-affected relative with SS (*χ* ^2^ = 34, *p* < 1 × 10^−8^, odds ratio = 4.4). Thus, SLE-unaffected relatives with “primary” SS were almost 5 times more likely to have a SLE-affected relative with “secondary” SS than were those relatives without “primary” SS (see Table 1).

## 4. Discussion

SLE and SS are autoimmune diseases that share clinical and serological features. The majority of Sjögren's patients have anti-Ro and about half of these same patients have anti-La antibodies. Both anti-Ro and anti-La also occur in those with SLE with less frequency, but anti-La is associated with lack of renal and central nervous system disease [20]. The diseases share clinical features as described and some patients present with both phenotypes (i.e., polyautoimmunity) [6, 21].

While much less research has been done on SS, thus far at least, the genetics of these two diseases are similar [11]. We found that among families recruited for the presence of SLE about 1% of SLE-unaffected relatives had SS, while none of over 1400 controls had this disease. Although it is well to remember that the controls were matched with the SLE patients, not the SLE-unaffected relatives, our data demonstrate that SLE and SS tend to occur in the same families.

We have defined SS in this study by the presence of dry eyes, dry mouth, and anti-Ro. This definition, of course, does not meet the usual research classification criteria [7] and is thus a potential weakness. On the other hand, we have used this same definition in this cohort previously with success [22]. Furthermore, combinations of clinical symptoms such as dry mouth, dry eyes, and painful mouth predict classification by the research criteria very well [23]. The Connective Disease Screening Questionnaire has a sensitivity of 85% and a specificity of 83% for Sjögren's syndrome [19]. Furthermore, a strategy combining this screening tool with autoantibody testing produced 95% sensitivity and 99% specificity along with 71% positive predictive value and 98% negative predictive value for RA [24]. Thus, these data support those in SS [23] where a screening questionnaire combined with autoantibody testing is useful for case finding. Nonetheless, we have likely misclassified some individuals, but there is no reason to suspect that such misclassification occurred in a way that biased the association result we found between “primary” and “secondary” SS in these families with SLE.

Our data also demonstrate that “secondary” SS among the SLE patients and “primary” SS are found within the same families such that individuals with “primary” SS were 4.4 times more likely to have a lupus-affected relative with “secondary” SS than were those without “primary” SS (Table 1). The data generated by the present work imply that there might be not only common genetic factors leading to these diseases but also common environmental (nongenetic) factors putting persons at risk (i.e., the autoimmune tautology) [25].

## Figures and Tables

**Table 1:** Distribution of secondary Sjögren's syndrome among relatives of those with systemic lupus erythematosus with and without primary Sjögren's syndrome.

	SLE-unaffected relatives	
	pSS	No pSS
SLE with sSS	18	530
SLE without sSS	53	6789

*χ* ^2^ = 34; *p* < 0.00000001; odds ratio = 4.4.

sSS: secondary Sjögren's syndrome; SLE: systemic lupus erythematosus.
